# Associations between Maternal and Infant Morbidities and sRAGE within the First Week of Life in Extremely Preterm Infants

**DOI:** 10.1371/journal.pone.0082537

**Published:** 2013-12-06

**Authors:** Lynette K. Rogers, Amanda E. Graf, Anisha Bhatia, Karen L. Leonhart, Reena Oza-Frank

**Affiliations:** 1 Center for Perinatal Research, The Research Institute at Nationwide Children’s Hospital, Columbus, Ohio, United States of America; 2 Department of Pediatrics, The Ohio State University, Columbus, Ohio, United States of America; University of Miami, United States of America

## Abstract

**Background:**

Soluble RAGE (sRAGE) has been associated with multiple inflammatory responses including maternal chorioamnionitis and preeclampsia. Analysis of umbilical cord blood levels have also indicated that sRAGE levels in the infant are affected by maternal inflammation. S100b is a ligand for RAGE and increases in circulating S100b levels are associated with poor neurological outcome in preterm infants. The objective of this study was to determine whether sRAGE or s100b levels in plasma samples from extremely preterm infants at the end of the first week of life were correlated with infant morbidities and whether sRAGE and s100b levels at this time point were still associated with maternal inflammation.

**Methods:**

Plasma samples were collected from 130 preterm infants (≤28 weeks) at days of life 5, 6, or 7. sRAGE and s100b levels were measured by ELISA and data were analyzed by Pearson’s correlation or Generalized Estimating Equations.

**Results:**

sRAGE was negatively correlated with development of sepsis (p=0.024), the FiO_2_ requirement of the infant at the time of sampling (p=0.030), as well as maternal preeclampsia (p=0.046), and positively correlated with maternal chorioamnionitis (p=0.006). s100b levels were positively associated with maternal chorioamnionitis (p=0.039). No correlations were observed with other infant morbidities.

**Conclusion:**

These data indicate that sRAGE could potentially be a biomarker of early severe inflammatory responses in the preterm infant. However, more studies are needed to confirm the present findings.

## Introduction

Nearly 12% of babies born in the United States are born prematurely before 37 completed weeks of gestation [1]. Many of these babies will require care in a neonatal intensive care unit and are at risk for a number of medical complications and morbidities. Of those babies born weighing less than 1500 grams, 25-50% will face some degree of long-term neurodevelopmental impairment [2]. The discovery of potential tools to identify those infants at highest risk for development of complications is an important research step toward the prevention of such morbidities. 

 Maternal infection and inflammation are known risk factors for premature birth [3,4]. As such, many premature infants are exposed to inflammatory stimuli even prior to birth, with additional inflammatory insults often occurring as a result of the subsequent resuscitation and Intensive Care Unit care necessary for survival [5]. Regardless of the source, inflammation leads to cell injury and death which then propagate further inflammatory responses, establishing a harmful cycle for a vulnerable infant. 

One mechanism by which cell injury contributes to a persistent inflammatory response is that of ligand/receptor mediated pathways. Dying cells release molecules collectively known as “Danger Associated Molecular Patterns” or DAMPs that are ligands for pattern recognition receptors, such as the Receptor for Advanced Glycation End Products (RAGE) [6]. Ligands for RAGE are diverse and include DAMPS such as high-mobility group box 1 (HMGB1) and s100s. Physiologically, RAGE can be either membrane bound to propagate signaling or in a soluble form (sRAGE), the result of alternative splicing or proteolytic cleavage [7–9]. Membrane bound RAGE is associated with increases in chemotaxis and propagation of inflammatory responses. sRAGE functions as a decoy receptor, binding ligand and preventing intracellular signaling, however more recently sRAGE has been shown to propagate inflammation by binding CD11b on the surface of leukocytes and activating NFkB-mediated pathways [10,11]. Consequently, the function of sRAGE depends upon the extracellular milieu and the severity of the inflammatory responses. 

The presence of ligands for RAGE may also be indicative of both RAGE activity and inflammatory stress. s100b, one particular ligand of interest for RAGE, is an acidic calcium-binding protein highly expressed in the nervous system. Its presence in extracellular fluids such as blood, cerebral spinal fluid, or urine is currently used as a biomarker of traumatic brain injury [12]. Recent studies have indicated that extracellular s100b signaling through activation of RAGE may be responsible for microglial migration and release of cytokines in the context of brain inflammation or injury [13]. 

Many potential biomarkers have been evaluated in the context of predicting risk for morbidities and poor outcome in preterm infants. Unfortunately, few have held up to scrutiny because of the multifactorial nature of these conditions. Neonatal morbidities, including sepsis and intraventricular hemorrhage (IVH) may occur within days after birth, but other morbidities such as late-onset sepsis, necrotizing enterocolitis (NEC), bronchopulmonary dysplasia (BPD), and periventricular leukomalacia (PVL) may occur weeks later. The etiologies of these conditions are complex and likely related to both pre- and post-natal risk factors. Thus postnatal biomarkers measured in the first week of life may demonstrate a predictive value. 

Consequently, we tested the hypothesis that plasma sRAGE or s100b levels in extremely preterm infants (≤28 weeks gestation) at 5-7 days of age would be associated with severity of early Respiratory Distress syndrome, incidence of early-onset sepsis, IVH, and later morbidities including NEC, BPD, and PVL. Additionally, we tested the association between exposure to maternal inflammation and sRAGE or s100b levels in the infant at the end of the first week of life. Because infants exposed to maternal chorioamnionitis, sepsis during hospitalization, severe BPD, surgical NEC, or severe IVH or PVL are at higher risk of moderate to severe neurodevelopmental impairment, prediction of both early onset and later morbidities in the neonatal population will provide additional data to assist in providing a long term prognosis for at risk infants. Eventually, identifying those infants most at risk will allow for adjustment of therapeutic strategy for both prevention and treatment.

## Methods

### Ethics Statement

Patients were enrolled by written informed consent of the parent(s) and recruited from The Ohio State University Wexner Medical Center (OSUWMC) and Nationwide Children’s Hospital (NCH) between April 2006 and July 2010. All studies were approved by the IRB of Nationwide Children’s Hospital, IRB# 05-00338.

### Patient enrollment

The original cohort included 473 infants ≤32 weeks gestation at birth. Seventy-one infants were multiples, sixty-five twins and six triplets. A total of twenty-nine were intrauterine growth restricted (IUGR) based on the growth curves established by Kramer et al.[14]. Infants were enrolled within the first 3 days of life. Infants with anomalies such as genetic disorders, congenital heart disease, or anomalies that would alter the standard of care for prematurity were excluded. Infants that died were also excluded because we were unable to collect samples for the given days and/or their outcomes could not be followed. Interim analyses for biomarkers of disease revealed that gestational age was most influential factor [15]. Because of the greater likelihood to develop diseases associated with prematurity and smaller range in gestational age, the present study was focused on a subset of infants ≤28 weeks gestation at birth. 

### Sample collection

 A single 0.5 mL sample of blood was drawn on postnatal day 5, 6 or 7 (day of birth is day 0). Day of life for sample varied due to day of enrollment (1, 2 or 3) and because samples could only be drawn every 48 hours for a total of three samples during a one week period by IRB approval. All samples were placed at 4° C immediately after collection, the cells were separated from the plasma and the plasma stored at -80° C until analysis. 

### Data Collection

Maternal and infant clinical data were collected from the infant’s chart from enrollment to hospital discharge. Data were collected on the specifics of birth, development of morbidities, and treatments. The diagnosis of BPD was defined as requiring respiratory support at 36 weeks corrected gestational age. Level of respiratory support in the infant was evaluated by the requirement of supplementary oxygen (FiO_2_). NEC was defined as Bell stage 2 or greater. For the purpose of this report, sepsis was defined as a positive culture of any body fluid within the first 10 days of life. Diagnosis of IVH required head ultrasonography with radiographic confirmation. Maternal chorioamnionitis was diagnosed histologically by pathology report, as stated in the infants chart. Preeclampsia was also designated as reported in the infants chart.

### Laboratory Analysis

Plasma sRAGE concentrations were measured using an ELISA based technology on a Meso Scale Discovery Sector Imager 2400 (cat. # N45ZA, Meso Scale Discovery, Rockville MD; *intra assay CV%, 4.8-6.2; inter-assay CV% 6.7-8.2* ). Plasma s100b levels were measured by sandwich ELISA (cat. # RD192090100R, BioVendor, Czech Republic; *intra-assay CV% 2.7-3.8; inter-assay 5.2-10.1*).

### Statistical Analysis

Generalized Estimating Equations (GEE) were used to determine the associations between sRAGE/s100b and FiO_2_, sepsis (within first 10 days of life), NEC, BPD, and IVH in the infant and maternal chorioamnionitis, preterm labor, and preeclampsia. GEE were used to account for clustering of multiple infants to a single mother, for example twins and triplets (15). Two sample t-test or Wilcoxon rank-sum test, where appropriate, was used to test effect differences for sRAGE and s100 between chorioamnionitis groups (Yes/No) and preeclampsia groups (Yes/No). Data were analyzed after log transformation of sRAGE and s100b values. Correlations between sRAGE and FiO_2_, sRAGE and s100b, and s100b and FiO_2_ were analyzed using Pearson’s correlation. The following independent variables were tested for confounding in all models: gestational age, gender, multiple birth, maternal age, maternal race, and mode of delivery (vaginal or cesarean section). Statistical significance was set at p<0.05 for single comparisons and with adjustment for multiple comparisons. All analyses were conducted using SAS version 9.3 (SAS Inc., Cary, NC, USA). 

## Results

 A total of 180 infants met inclusion criteria. However, only 130 infants had plasma samples from the dates specified. Informed consent of the mother prior to day of life 3 and acuity of the infant’s medical status (making sample collection unethical) were the most limiting variables. The demographic data from enrolled participants included in our analyses are presented in Table 1. Of the 130 infants included, 19 exhibited IUGR. At the time of sample collection, 47 infants were on mechanical ventilation, 80 were on nasal continuous positive airway pressure, 2 were on nasal cannula, and 1 was on room air. Of the 46 infants diagnosed with IVH, 40 were grade I or II, 6 were grade III or IV. Of the 46 with IVH, 9 developed PVL. A total of 20 infants developed NEC, 11 were managed medically and 9 required surgical intervention. Because of limited sample size, IVH and NEC were considered without severity. 

**Table 1:** Summary of Patient Demographics and Clinical Characteristics.

	*n=130*
Infant	
Gestational age, mean (SD)	25.5 (1.3)
Weight, mean (SD)	815.2 (200.1)
Male (%)	77 (59.2)
Multiple birth (%)	29 (22.3)
Sepsis (%)	17 (13.1)
Necrotizing enterocolitis (%)	20 (15.4)
Bronchopulmonary dysplasia (%)	81 (623.3)
Intraventricular hemhorrage (%)	46 (35.4)
Maternal	
Age, mean (SD)	26.0 (5.6)
Race (%)	
Caucasian	100 (76.9)
African American	28 (21.5)
Other	2 (1.5)
Cesarean section	93 (71.5)
Chorioamnionitis (%)	63 (48.5)
Preterm labor (%)	47 (37.0)
Preeclampsia (%)	20 (15.9)

SD=standard deviation

**Figure 1:**
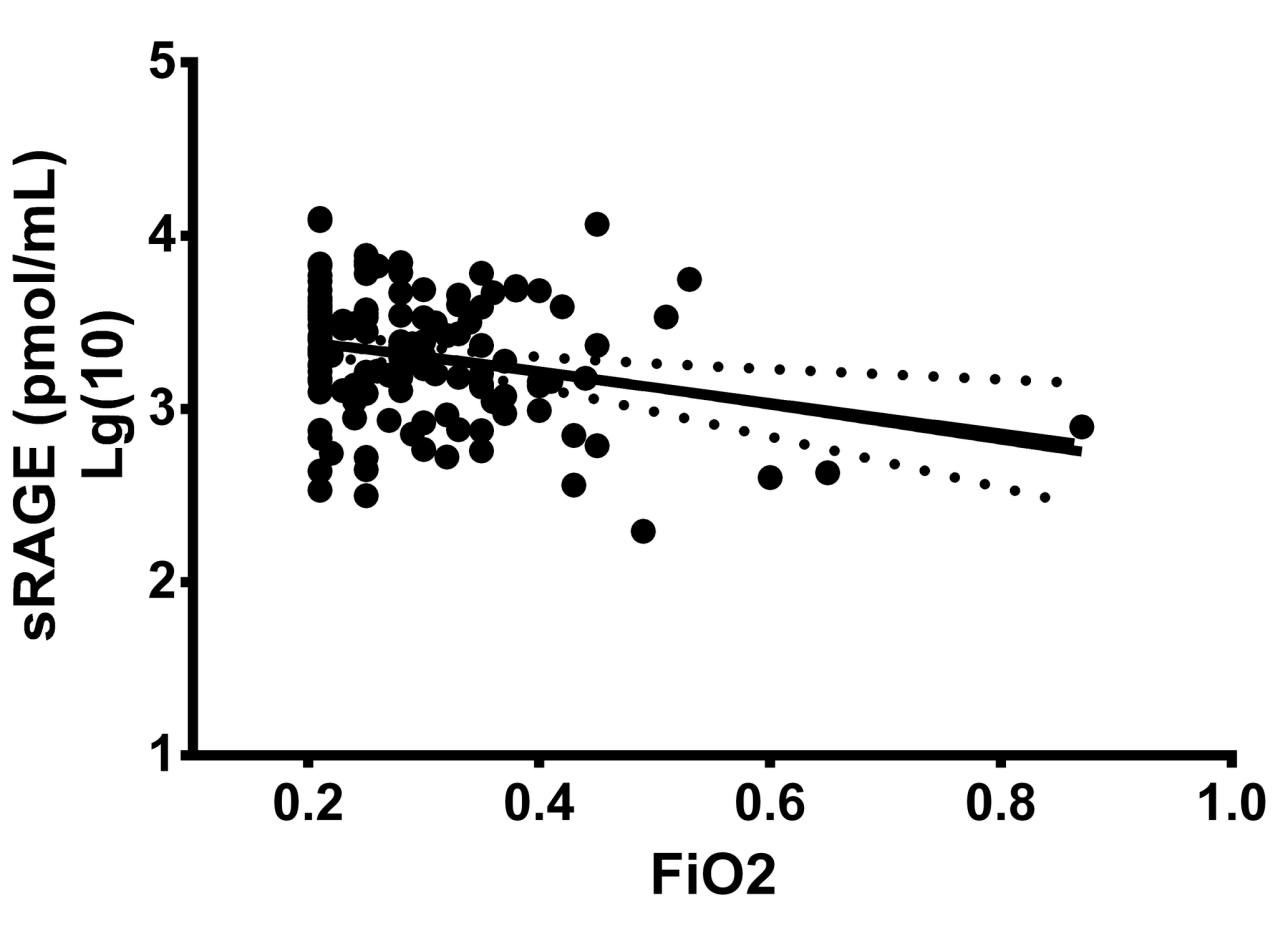
sRAGE levels were measured in infant plasma taken between 5-7 days of life. Data were analyzed by Pearson’s correlation. sRAGE content negatively correlated with the FiO_2_ the infant was receiving at the time of sampling (r=-0.254, p=0.0038).

For all regression models, maternal age and race were not significantly associated with the outcomes of interest, thus all models were adjusted for gestational age, gender, multiple birth, and mode of delivery. sRAGE and s100b levels were negatively associated with presence and severity of early onset neonatal morbidities at the time of sample collection including sepsis and respiratory distress syndrome. A negative correlation was found between sRAGE levels and early onset culture-positive sepsis (Table 2). Level of respiratory support in the infant was evaluated by the requirement of supplementary oxygen (FiO_2_). A negative correlation between sRAGE and FiO_2_ levels was observed at the time of sampling in unadjusted analyses. (Figure 1, Table 3) and in the adjusted analyses (Table 2). s100b levels were also negatively associated with FiO_2_ levels in unadjusted analyses , but these associations did not remain significant in adjusted analyses (Table 2). Contrary to our initial hypothesis, sRAGE and s100b levels were not significantly associated with later diagnosis of infant morbidities. 

**Table 2:** Associations between sRAGE, s100b and FiO_2_, sepsis, chorioamnionitis, NEC, BPD, fetal distress, feeding intolerance, and IVH using GEE.

	sRAGE			S100b		
**Infant**						
	β	SE	p-value	β	SE	p-value
FiO_2_	-0.066	0.03	**0.030**	-0.053	0.05	0.260
Sepsis	-1.683	0.75	**0.024**	-1.362	0.96	0.154
Necrotizing enterocolitis	0.342	0.74	0.642	0.582	0.95	0.540
Bronchopulmonary dysplasia	-0.401	0.51	0.436	-0.443	0.77	0.565
Intraventricular hemhorrage	-0.188	0.54	0.729	-1.609	0.85	0.059
**Maternal**						
	β	SE	p-value	β	SE	p-value
Preeclampsia	-1.565	0.79	**0.046**	-0.803	1.05	0.445
Preterm labor	0.928	0.51	0.067	0.546	0.76	0.473
Chorioamnionitis	1.657	0.61	**0.006**	1.700	0.82	**0.039**

All analyses adjusted for gestational age, gender, multiple birth, and mode of delivery; sRAGE and s100b values were log transformed for analyses.

**Table 3:** Pearson’s correlations between sRAGE and s100b or the infant FiO_2_ at the time of sampling.

	sRAGE		s100b	
	r	p-value	r	p-value
FiO_2_	-0.254	**0.0038**	-0.229	**0.011**
s100b	0.228	**0.015**	.	.

Correlations (Pearson’s).

sRAGE and s100b values were log transformed for analyses.

When examining the influence of maternal inflammatory status on neonatal levels at this time point, infant sRAGE levels were positively associated with maternal chorioamnionitis (β=1.657, p=0.006) (Table 2) and negatively associated with preeclampsia (β=-1.565, p=0.046) (Table 2). s100b levels were positively associated with chorioamnionitis (β=1.700, p=0.039) (Table 2). As expected, sRAGE plasma concentrations were associated with s100B concentrations (Table 3).

## Discussion

sRAGE levels have been associated with increased inflammation in both the mother and the infant at the time of birth [16,17]. In human preterm neonates exposed to maternal chorioamnionitis or funisitis, sRAGE levels are lower in both tracheal aspirate samples and umbilical cord blood when compared to control infants [16,18]. In fact, the more severe the maternal inflammation the lower the cord blood sRAGE levels [16]. However, little is known about sRAGE plasma or tracheal aspirate levels throughout gestation or in a healthy term infant. Unlike previous investigations of umbilical cord blood, we investigated infant plasma sRAGE levels at the end of the first week of life to determine whether expression levels of sRAGE could be a predictive biomarker for infant morbidities. 

 Data regarding sRAGE changes in response to postnatal insults include increases in sRAGE levels seen in bronchoalveolar lavage or tracheal aspirate samples with acute lung injury, including exposure to hyperoxia in rats [11], mice [19], and in humans [20]. However, correlations between later infant morbidities such as sepsis, NEC, BPD, IVH, or PVL and sRAGE have not been reported. 

Severe inflammatory insults such as neonatal sepsis can result in widespread tissue injury and further inflammation. As cellular injury is occurring, both sRAGE and ligands for RAGE are being released [21,22]. The free sRAGE is likely binding to ligands but because it is extracellular and functions as a decoy receptor to mediate inflammatory responses [23]. Thus, a negative association between sRAGE and development of sepsis may be an indicator of severity of inflammation. Our findings support the hypothesis that acute infection or inflammation leads to increases in sRAGE levels and RAGE ligands which rapidly bind but prevent downstream signaling. 

 We also observed a negative correlation between sRAGE levels and the amount of oxygen required by the infant for respiratory support. FiO_2_ requirement in this study was evaluated as a marker for severity of early respiratory illness. This particular measure is also useful because those infants requiring the most supplemental oxygen for management of respiratory illness are also potentially receiving the highest degree of additional inflammatory insults from oxidant stress. This data is similar to that reported in rodents exposed to hyperoxia during the first week of life [24], and in humans with acute lung injury [25,26]. Alternatively, intracellular RAGE signaling is important for normal lung development and alveolarization [27–29]. In the face of increased sRAGE levels, it is reasonable to speculate that the infant born to an infected mother would upregulate the transcription of RAGE to provide the necessary receptor-mediated signaling for lung growth.

s100b is a ligand of RAGE, is highly expressed in neurological tissues, and elevated plasma levels have been associated with neurological injuries [30]. Specifically, s100b has been shown to be associated with diagnosis of brain injury in preterm infants [27,28]. We did not observe correlations between sRAGE or s100b levels and development of later onset inflammatory morbidities such as late onset sepsis, BPD, NEC, IVH, or PVL. Taken together these findings would suggest that sRAGE and s100b levels may be useful as markers for active inflammation at the time of sampling but their predictive value for risk of future morbidities remains unclear. 

As previously reported, sRAGE levels in amniotic fluid in women with a diagnosis of preterm labor or PPROM and intrauterine infection are elevated compared to women without infection [17,31]. In the same light, s100b protein levels were positively associated with chorioamnionitis similar to sRAGE protein levels. Our data indicate a positive association rather than a negative association between sRAGE levels and maternal chorioamnionitis upon conclusion of the first week of life. Our best explanation is the timing of the sample collection. Nearing the end of the first week of life many of the maternal influences have resolved and the infant is responding to current stimuli. 

 Several studies have identified an association between maternal preeclampsia and sRAGE levels [32,33]. Whether increase in sRAGE expression is involved in initiation of injury or is a product of ongoing pathology is not well understood. However, pregnancy alone causes increases in RAGE expression, which is further increased in the context of preeclampsia [32]. Most previous reports are focused on the maternal plasma, umbilical cord blood, or placental and fetal tissue. We observed a negative correlation between maternal preeclampsia and infant plasma [32] (Table 3) which may represent resolution of maternally induced inflammatory responses. 

These studies were designed to explore whether sRAGE and/or s100b would be viable candidates as biomarkers for development of later infant morbidities. The greatest weakness to these studies is the small number of positive disease cases making it statistically difficult to discern differences in sRAGE associated with maternal and infant morbidities, such as the severity of chorioamnionitis or preeclampsia in the mother and PVL or NEC in the infants. Additional factors such as administration of maternal steroids that may impact these associations were also difficult to assess due to the majority of women (n=117) having received some form of antenatal steroids. Another limitation is the exploratory nature of the current study. Our original cohort investigation was observational and was not powered for any specific associations. As a secondary analysis of the observational study, these investigations were strictly exploratory and not necessarily powered for conclusions. Our data project interesting trends, supporting the need for larger multicenter studies to determine efficacy of these molecules as predictive tools for identification of the highest risk infants.

In conclusion, sRAGE and s100b protein levels in extremely preterm infants near the end of the first week of life were negatively correlated with active inflammatory processes in the neonates including respiratory distress syndrome, sepsis, and IVH. These data indicate that sRAGE could potentially be a biomarker of early severe inflammatory responses in the preterm infant. Consequently, more studies are needed to determine whether early measurement of sRAGE and/or s100b may be a useful biomarker for predicting risk of morbidities in preterm infants. 
